# Immune signature as predictive marker for response to checkpoint inhibitor immunotherapy and overall survival in melanoma

**DOI:** 10.1002/cam4.3710

**Published:** 2021-01-15

**Authors:** Franziska K. Krebs, Emily R. Trzeciak, Sophia Zimmer, Deniz Özistanbullu, Heidrun Mitzel‐Rink, Markus Meissner, Stephan Grabbe, Carmen Loquai, Andrea Tuettenberg

**Affiliations:** ^1^ Department of Dermatology University Medical Center of the Johannes Gutenberg‐University Mainz Germany; ^2^ German Cancer Consortium (DKTK) Partner Site Mainz/Frankfurt am Main Mainz Germany; ^3^ Department of Dermatology, Venereology and Allergology Johann Wolfgang Goethe University Frankfurt Germany

**Keywords:** checkpoint inhibitor, immunotherapy, malignant melanoma, myeloid‐derived suppressor cells, platelets

## Abstract

**Background:**

Malignant melanoma is an immunogenic skin cancer with an increasing global incidence. Advanced stages of melanoma have poor prognoses. Currently, there are no reliable parameters to predict a patient's response to immune checkpoint inhibitor (ICI) therapy.

**Methods:**

This study highlights the relevance of a distinct immune signature in the blood for response to ICI therapy and overall survival (OS). Therefore, the immune cell composition in the peripheral blood of 45 melanoma patients prior to ICI therapy was analyzed by flow cytometry and complete blood count.

**Results:**

Responders to ICI therapy displayed an abundance of proliferating CD4^+^ T cells, an increased lymphocyte‐to‐monocyte ratio, a low platelet‐to‐lymphocyte ratio, low levels of CTLA‐4^+^ Treg, and (arginase 1^+^) polymorphonuclear myeloid‐derived suppressor cells (PMN‐MDSC). Nevertheless, non‐responders with similar immune cell compositions also benefited from therapy displaying increased long‐term OS.

**Conclusions:**

Our study demonstrated that the observed immune signature in the peripheral blood of melanoma patients prior to treatment could identify responders as well as non‐responders that benefit from ICI immunotherapies.

## Graphical abstract

In this study, we offer insight into how immune cell composition in the peripheral blood before beginning immune checkpoint inhibitor (ICI) therapy differs in responders and non‐responders to ICI. Notably, non‐responders with an immune cell composition comparable to responders displayed a prolonged overall survival (OS). This newly characterized immune signature aids in improving the identification of patients (responders and non‐responders) that would benefit from ICI treatment.

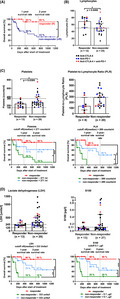

## 1 INTRODUCTION

In 2018, the global incidence of melanoma was 287,723 cases and is predicted to rise. Worldwide, malignant melanoma represents 2.6% of newly diagnosed cancer cases (ASR, age‐standardized incidence rate) and is one of the most fatal types of skin cancers.^1^ One of the current strategies to treat late‐stage melanoma is the application of immune checkpoint inhibitors (ICI). ICI block co‐inhibitory receptors, such as CTLA‐4 (cytotoxic T‐lymphocyte–associated protein 4) and PD‐1 (programmed cell death protein 1), on T cells resulting in the constant activation and proliferation of T effector cells.^2^ Since the anti‐CTLA‐4 ICI Ipilimumab was approved for the treatment of melanoma in 2011, the number of ICI and ICI treatment approved tumor entities has steadily risen.^3^ Hence, there is an increasing demand to understand ICI response patterns. Responders exhibit either fast, slow, or delayed responses with initial pseudo‐progression, whereas non‐responders exhibit progressive disease. Delayed responders can start to respond as late as the second staging, and thus, these patients are often initially incorrectly classified as non‐responders. Until then, patients are at risk of developing ICI‐induced life‐threatening immune‐related adverse events (irAE), such as colitis, and face extensive therapy costs.^4^

The objective of this study was to identify an immune signature in the peripheral blood of stage III/IV melanoma patients before the start of treatment favoring response to ICI therapy and long‐term overall survival (OS). So far, it is known that ICI release the brakes on T‐cell proliferation and possibly deplete regulatory T cells (Treg) via antibody‐dependent cellular cytotoxicity (ADCC) and phagocytosis (ADCP).^5^, ^6^, ^7^ In addition, current publications highlight the immunosuppressive role of myeloid‐derived suppressor cells (MDSC) in resistance to cancer immunotherapies and investigate MDSC as therapeutic targets.^8^

However, knowledge about a favorable immune signature in the peripheral blood leading to ICI therapy response and long‐term survival is limited. To meet this need, we compared percentages of key immune cells (T cells, Treg, and MDSC) in the peripheral blood of responders and non‐responders before the start of therapy, evaluated their impact on immune response by using functional data (checkpoint molecules, effector cytokines, and cell proliferation), and analyzed whether this affected the long‐term OS of responders and non‐responders.

Therefore, we recruited melanoma patients before beginning ICI therapy (anti‐CTLA‐4, anti‐PD‐1, anti‐CTLA‐4 + anti‐PD‐1) and analyzed the immune cell composition and function of peripheral mononuclear blood cells using flow cytometry. Complete blood count provided additional data on platelet count and the prognostic melanoma biomarkers, S100 and lactate dehydrogenase (LDH).

In this study, a favorable immune signature leading to ICI therapy response consisted of an abundance of proliferating CD4^+^ T cells, a high lymphocyte‐to‐monocyte ratio, a low platelet‐to‐lymphocyte ratio, low levels of CTLA‐4^+^ Treg, and decreased percentages of (arginase 1^+^) PMN‐MDSC. Of note, a similar immune signature in a subgroup of “clinical” non‐responders led to long‐term survival in this cohort. As a result, this study highlights that identifying a distinct immune signature in the peripheral blood may hold the key to long‐term survival of melanoma patients.

To sum up, this study confirms known prognostic indicators (i.e., LDH and S100), gives evidence to potential new ones (i.e., platelets), and provides a comprehensive overview of a favorable immune signature that can identify responders. Interestingly, we also discovered that a subgroup of “clinical” non‐responders, who display an immune signature similar to responders, benefit from ICI therapy by exhibiting long‐term overall survival.

## 2 MATERIALS AND METHODS

### 2.1 Patient samples

This open‐label study recruited 45 patients with unresectable advanced stage malignant melanoma at two study centers, the Departments of Dermatology of the University Medical Center Mainz and the Johann Wolfgang Goethe University, between April 2014 and July 2017. The study follow‐up ended in January 2018. We included patients over the age of 18 that were about to start the following ICI therapies: Ipilimumab (anti‐CTLA‐4 ICI, Yervoy®), Nivolumab (anti‐PD‐1 ICI, Opdivo®), Pembrolizumab (anti‐PD‐1 ICI, Keytruda®), or Ipilimumab/Nivolumab (combination therapy). Patients with infectious diseases were excluded from this study. The clinical endpoints were comprised of the patient's death (primary), the discontinuation of the study due to irAE, non‐compliance, or withdrawal of consent (secondary). The study protocol (837.029.05 (4687)) was approved by the local Ethics Committee of Rhineland‐Palatinate and Hessen (Landesärztekammer). All procedures in this study involving human participants were performed in accordance with the 1964 Declaration of Helsinki and its later amendments. Informed written consent was obtained from every study participant. Blood samples of eight unmatched healthy donors served as controls.

This study analyzed the peripheral blood of patients before the start of treatment to identify immune cell compositions that favor response to ICI therapy and long‐term OS. Patients were divided into non‐responders (progressive disease) and responders (complete response, partial response, or stable disease) at the second staging six months after the start of therapy according to iRECIST criteria.^9^ Patients received either 3 mg/kg body weight Ipilimumab monotherapy intravenously (IV) 4 times within a 3‐week interval, Nivolumab monotherapy with a dosage of 3 mg/kg IV every 2 weeks, or Ipilimumab/Nivolumab combination therapy, which consisted of 1 mg/kg Nivolumab IV and subsequently 3 mg/kg Ipilimumab IV 4 times within a 3‐week interval. Combination therapy was followed by Nivolumab monotherapy. The Pembrolizumab monotherapy contained 2 mg/kg IV infusion, which was conducted every 3 weeks.

### 2.2 Sample collection

Two 20 ml heparin syringes with peripheral blood and one Sarstedt Heparin‐Li Plasma monovette were collected prior to therapy. Peripheral blood mononuclear cells (PBMC) and plasma were obtained by density gradient centrifugation (Biocoll, Merck). PBMC were frozen in human‐albumin (HA), 20% Behring low‐salt infusion solution (200 g/L, CSL Behring GmbH) containing 10% dimethyl sulfoxide (DMSO, D2650, Merck), and stored at −80°C until further analysis. Samples were frozen for a maximum duration of three years. Complete blood count provided data on S100, LDH, platelet count, and platelet‐to‐lymphocyte ratio.

### 2.3 Flow cytometry

Flow cytometry was conducted on a BD LSR II using BD Diva 6 and 8. Flow cytometric data were analyzed using Cytobank.^10^ Cryopreserved patient PBMC were thawed, washed with flow cytometry buffer, and directly stained for flow cytometric analysis without prior stimulation. Flow cytometry buffer contained 1 L phosphate‐buffered saline (PBS) pH 7.2 (Gibco, #20012027), 25 ml HA, 2 ml EDTA solution pH 8 (0.5 M) (AppliChem GmbH), and 100 µl Privigen 100 mg/ml (CSL Behring GmbH). Intracellular staining (arginase 1, granzyme B, IFN‐γ) was performed using BD Cytofix/Cytoperm^TM^ Solution Kit (#554714, BD), while intranuclear staining (Foxp3, Ki‐67) was performed using eBioscience^TM^ Foxp3/Transcription Factor Staining Buffer Set (#00‐5523‐00) according to the manufacturer's instructions. The expression of the checkpoint molecules, CTLA‐4 and PD‐1, was analyzed on the surface of cells. We distinguished MDSC based on the recommendations made by Mandruzzato et al.^11^: CD14^+^CD15^−^CD33^+^HLA‐DR^low^ monocytic/mononuclear (M)‐MDSC, CD15^+^CD33^+^ polymorphonuclear (PMN)‐MDSC, and HLA‐DR^low^CD11b^+^CD33^+^ early (E)‐MDSC (Figure S2A).

We used the following antibodies: Arginase 1 (#IC8026A, R&D), CD3 (#100248, BioLegend), CD4 (#130‐100‐454, Miltenyi), CD8 (#555369, #563676, BD), CD11b (#555388, BD; #130‐081‐201, Miltenyi), CD14 (#21620145, ImmunoTools), CD15 (#560827, BD; #21810156, ImmunoTools), CD25 (#555433, BD), CD33 (#561157, BD), CD127 (#351306, BioLegend), CTLA‐4 (#ABIN2144728, antibodies‐online; #130‐097‐684, Miltenyi), Foxp3 (#320208, BioLegend), GARP (#130‐103–820, Miltenyi), Granzyme B (#561151, BD), HLA‐DR (#307618, BioLegend; #130‐095‐295, Miltenyi), IFN‐γ (#554551, BD), Ki‐67 (#130‐100‐330, Miltenyi), and PD‐1 (#130‐096‐166, Miltenyi). We excluded doublets, debris, and dead cells (fixable viability dye, #65‐0866‐14, Thermo Fisher Scientific) from analysis.

### 2.4 Survival analysis and definition of cutoffs

Survival analysis was performed by using the Kaplan‐Meier method and GraphPad Prism version 8.4.3 for Windows, GraphPad Software, www.graphpad.com. Survival curves were compared using the Log‐rank (Mantel‐Cox) test. Patient survival was measured starting at the day of first drug administration (day 0) till the date of death or last contact. 14 non‐responders and 12 responders were depicted as censored data points in the Kaplan‐Meier curves, which indicate the last contact to a patient with no follow‐up data or date of death.

Within the non‐responder population, some patients showed an immune signature, which more closely resembled the responder population. Therefore, we determined cutoffs distinguishing between the two groups within non‐responders and compared their OS. These two populations were defined as being above or below the designated cutoff. Cutoffs were individually defined for each parameter and designated as the median of the corresponding non‐responder population. With these cutoffs, we could specifically define and analyze the OS and immune signature of the two unique non‐responder populations in further detail.

### 2.5 Statistical analysis

Statistical analysis was performed using GraphPad Prism version 8.4.3 for Windows, GraphPad Software, www.graphpad.com. Data were analyzed using a two‐tailed Mann‐Whitney test corrected for multiple comparisons with Dunn's test. Significant statistical differences between groups were highlighted in plots by the corresponding *p* value and asterisks. Plots without asterisks indicate that there were no significant differences. Statistical significance was defined as *p* < 0.05 (*), *p* < 0.01 (**), and *p* < 0.001 (***). Since the graphs show the results of pooled data, different ICI treatments were color‐coded: anti‐CTLA‐4 (Ipilimumab) in black, anti‐PD‐1 (Nivolumab, Pembrolizumab) in blue, and anti‐CTLA‐4/PD‐1 combination therapy in red. The number of patients per column may differ because parameters (i.e., Ki‐67) were analyzed toward the end of the study, patient samples were used up, or the complete blood count of patients was not available.

## 3 RESULTS

### 3.1 Patient characteristics

45 patients with unresectable, late‐stage, malignant melanoma were recruited, of which 60% were about to begin anti‐CTLA‐4, 24% anti‐PD‐1, and 16% anti‐CTLA‐4/PD‐1 combination therapy (Table 1). The average study participant was male, older than 60 years, diagnosed with stage IV melanoma BRAF wild type (73% of patients), and had received no previous systemic treatments prior to the study.

**TABLE 1:** Patient characteristics and treatments

Patient characteristics	n	%
Patients	45	100
Gender		
Male	27	60
Female	18	40
Median age, years (range)	70 (27–86)	
<65 years	19	42
≥65 years	26	58
Melanoma stage		
Unresectable melanoma stage IIIC	3	7
Unresectable melanoma stage IV	42	93
Treatments during the study		
Ipilimumab	27	60
Ipilimumab/Nivolumab	7	16
Pembrolizumab	9	20
Nivolumab	2	4
Response to treatments during the study		
Therapy success	13	29
Complete response	1	2
Partial response	9	20
Stable disease	3	7
Therapy failure	29	64
Unknown outcome	3	7
Treatments prior to the study		
Systemic treatment		
Chemotherapy	1	2
BRAF and MEK inhibitors	9	20
Checkpoint inhibitors	3	7
Number of systemic treatments		
0	32	71
1	9	20
2	4	9
Radiotherapy		
Cerebral radiation	3	7
Peripheral radiation	5	11
Adjuvant immunotherapy		
Adjuvant interferon immunotherapy	2	4
Mutanome Engineered RNA Immunotherapy (MERIT)	3	7
Transarterial chemoembolization (TACE)	1	2
Electro cancer therapy (ECT)	1	2

Note

The percentages refer to the total number of patients (n = 45) and are rounded. Prior to the study, patients received up to two previous systemic treatments. Three patients had already been treated with different immune checkpoint inhibitors: One patient received Nivolumab, the other Ipilimumab, and the third one received Ipilimumab followed later by Pembrolizumab.

Abbreviations: BRAF, B‐Raf proto‐oncogene kinase; MEK, mitogen‐activated protein kinase kinase.

**FIGURE 1:**
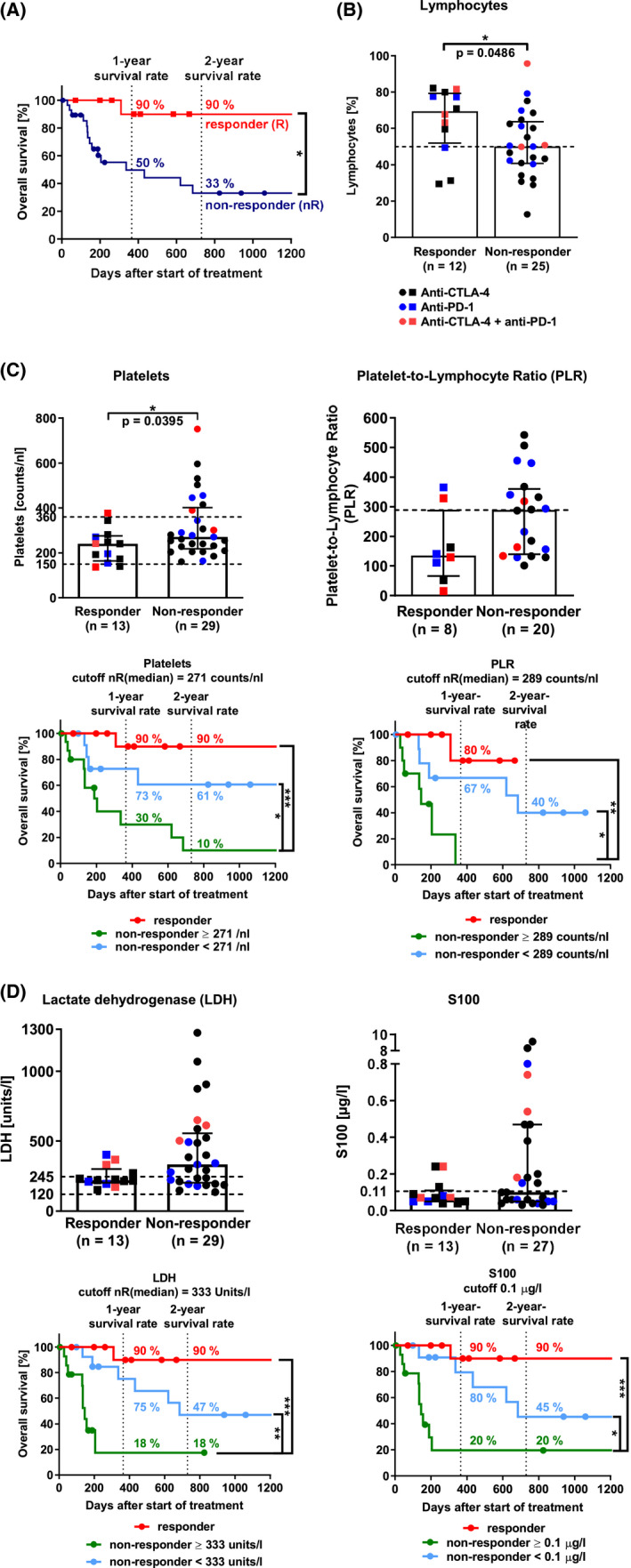
Immunomonitoring of lymphocytes, monocytes, platelets, and the melanoma biomarkers, S100 and LDH. (A) Overall survival of responders and non‐responders to immune checkpoint inhibitor (ICI) therapy (Log‐rank (Mantel‐Cox) test, *p* = 0.0253 *). (B) Lymphocytes. The bar diagrams depict the percentage of lymphocytes in viable cells. Lymphocytes were identified in FSC‐A/SSC‐A plots via flow cytometry. Doublets and dead cells were excluded from analysis. The dashed line marks the median non‐responder value (two‐tailed Mann‐Whitney test, *p* = 0.0486 *). (C) Platelets, platelet‐to‐lymphocyte ratio (PLR), and survival analysis. Complete blood count (CBC) provided lymphocyte and platelet count to calculate PLR. The dashed lines mark the normal range (two‐tailed Mann‐Whitney test, *p*(platelets) = 0.0395 *, *p*(PLR) = 0.0552; Log‐rank (Mantel‐Cox) test, survival analysis, *p*(platelets, nR >271 vs. nR <271) = 0.0251 *, *p*(platelets, nR >271 vs. R) = 0.0006 ***, *p*(PLR, nR >289 vs. R) = 0.0028 **, *p*(PLR, nR >289 vs. nR <289) = 0.0353 *). (D) LDH, S100, and survival analysis. CBC provided data on LDH and S100 levels. The dashed lines mark the normal range (two‐tailed Mann‐Whitney test, *p*(LDH) = 0.0569, *p*(S100) = 0.2532); Log‐rank (Mantel‐Cox) test, survival analysis, *p*(LDH, nR >333 units/L vs. nR <333 units/L) = 0.0063 **, *p*(LDH, nR >333 units/L vs. R) = 0.0002 ***; *p*(S100, nR >0.1 μg/L vs. nR <0.1 μg/L) = 0.0188 *, *p*(S100, nR >0.1 μg/L vs. R) = 0.0004 ***). Medians with interquartile range. CTLA‐4, cytotoxic T‐lymphocyte–associated protein 4; ICI, immune checkpoint inhibitors; LDH, lactate dehydrogenase; nR, non‐responder; PD‐1, programmed cell death protein 1; R, responder; S100, S100 protein

This study confirmed that about one in three patients responds to ICI therapy.^12^ Most often, the response observed was a partial response. Only one patient responded completely. Taken together, patients with clinical benefit of ICI therapy (responders) lived significantly longer than non‐responders (Figure 1A, *p* = 0.0253 *). The median OS of non‐responders was 336 days. The median OS of responders was undefined. The 1‐year and 2‐year OS of responders were about twice and three times higher than that of non‐responders, respectively. After 684 days, 50% of all study participants had died or were lost to follow‐up regardless of response to ICI therapy. In this study, immune‐related adverse events (irAE) were not associated with ICI therapy response (Table S1).^13^ One third of patients developed irAE, most commonly colitis. 50% of these patients discontinued therapy. Ipilimumab displayed the highest irAE incidence and discontinuation percentage.

Altogether, these data highlight that ICI therapy response is the key factor for long‐term OS. Since ICI target T cells, we hypothesized that immune cell composition in the peripheral blood of patients prior to treatment may also affect ICI therapy response and OS.

### 3.2 High lymphocyte and low platelet count indicate ICI therapy response

To gain further insight into the immune cell compositions beneficial to ICI therapy, we first analyzed lymphocytes and monocytes in the peripheral blood of patients via flow cytometry. Both cell populations are relevant to ICI therapy response, as T‐lymphocytes are the main target of ICI, and monocytes include immunosuppressive cells like MDSC. Responders had significantly higher percentages of lymphocytes in the peripheral blood than non‐responders (Figure 1B, *p* = 0.0486 *). In addition, data indicate that responders had double the lymphocyte‐to‐monocyte ratio compared to non‐responders (Figure S1A).

Since the latest studies highlight the importance of activated platelets in cancer progression, metastasis, and poor prognosis,^14^ we assessed platelet count and platelet‐to‐lymphocyte ratio (PLR) by complete blood count. In this study, responders had a significantly lower platelet count than non‐responders (Figure 1C, *p* = 0.0395 *). The platelet count stayed within normal range (150–360 counts/nl) in 79% of patients. Responders had also less than half the median PLR of non‐responders (*p* = 0.0552). Interestingly, within the non‐responder population, a subgroup of patients showed a PLR which more closely resembled the responder population. Therefore, we created “cutoffs” (see Material and Methods) to differentiate between these two groups within the non‐responders and compared their OS. The cutoff of a parameter was defined as its respective median value in non‐responders. Non‐responders with a platelet count and PLR above the designated cutoff (median non‐responder level) had a significantly reduced OS than non‐responders below the cutoff (Figure 1C, *p*(platelets) = 0.0251 *, *p*(PLR) = 0.0353 *). Firstly, non‐responders with platelet counts below the cutoff had twice the 1‐year OS than non‐responders above. Secondly, none of the non‐responders with a PLR above the cutoff lived longer than one year after the start of ICI therapy. These findings suggest that platelet counts and PLR within normal range may be potential parameters for good prognosis in terms of OS, especially in “clinical” non‐responders to ICI therapy.

Based on the information gained by the application of cutoffs, we decided to implement this strategy for all following analyses. Thereby, we could analyze the OS and immune signature of the two unique non‐responder groups. A good prognosis, in terms of OS and metastasis,^15^, ^16^ is also indicated by low levels of the malignant melanoma prognostic biomarkers, lactate dehydrogenase (LDH), and S100. We found that responders had lower median levels of LDH than non‐responders (Figure 1D, *p* = 0.0569). 69% of responders had LDH levels within the normal range (120–245 units/L), but only 38% of non‐responders did. In this study, the median S100 levels in responders and non‐responders were comparable (*p* = 0.2532). However, responders tended to have S100 levels below the upper normal S100 value (0.1 μg/L). Non‐responders below the S100 and LDH cutoff not only lived significantly longer than non‐responders above the corresponding cutoffs (*p*(LDH) = 0.0063 **, *p*(S100) = 0.0188 *) but also had a fourfold higher 1‐year OS. Our data suggest that S100 and LDH levels far above their normal ranges indicate non‐response to ICI therapy and reduced long‐term OS in non‐responders.

In summary, our results suggest that parameters of complete blood count, like lymphocytes, platelet count, and PLR, in combination with the melanoma biomarkers, LDH and S100, may present as potential convenient and cost‐effective indicators for ICI therapy outcome and long‐term OS of non‐responders.

### 3.3 Abundance of proliferating CD4^+^ T cells suggests ICI therapy response

T effector cells represent the target ICI cell population. ICI block checkpoint molecules, sustain T‐cell activation, and proliferation and thereby boost the adaptive immune response against cancer antigens.^2^ Therefore, we measured the percentage of T cells, checkpoint molecule expression (CTLA‐4, PD‐1), proliferation (Ki‐67), and effector cytokine production (IFN‐γ, granzyme B) of CD4^+^ and CD8^+^ T cells in patient PBMC prior to therapy via flow cytometry.

**FIGURE 2:**
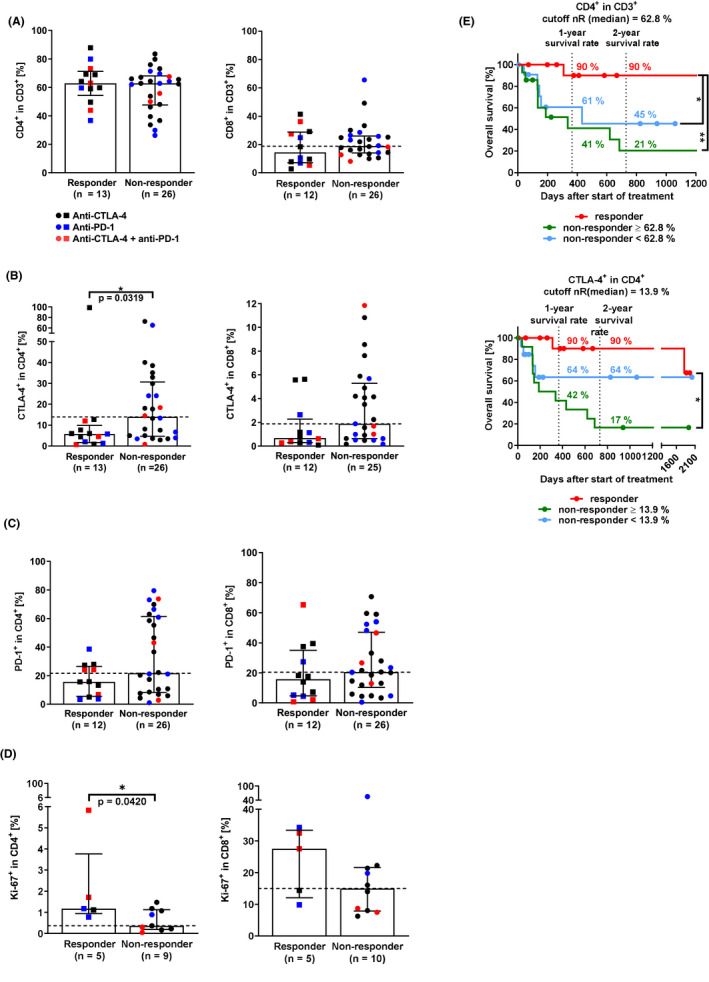
Immunomonitoring of CD4^+^ and CD8^+^ T cells in the peripheral blood. (A) CD4^+^ and CD8^+^ T cells (two‐tailed Mann‐Whitney test, *p*(CD4^+^ T cells) = 0.6274, *p*(CD8^+^ T cells) = 0.3274). (B) CTLA‐4^+^ CD4^+^ and CD8^+^ T cells (*p*(CTLA‐4^+^CD4^+^ T cells) = 0.0319 *, *p*(CTLA‐4^+^CD8^+^ T cells) = 0.0615). (C) PD‐1^+^ CD4^+^ and CD8^+^ T cells (*p*(PD‐1^+^CD4^+^ T cells) = 0.1550, *p*(PD‐1^+^CD8^+^ T cells) = 0.3431). (D) Proliferating (Ki‐67^+^) CD4^+^ and CD8^+^ T cells (*p*(Ki‐67^+^CD4^+^ T cells) = 0.0420 *, *p*(Ki‐67^+^CD8^+^ T cells) = 0.2065). (E) Survival analysis of patients depending on CD4^+^ and CTLA‐4^+^CD4^+^ T cells (Log‐rank (Mantel‐Cox) test, CD4^+^ T cells, *p*(nR >62.8% vs. R) = 0.0063 **, *p*(nR <62.8% vs. R) = 0.0251 *; CTLA‐4^+^CD4^+^ T cells, *p*(nR >13.9% vs. R) = 0.0025 **). Medians with interquartile range. CTLA‐4, cytotoxic T‐lymphocyte–associated protein 4; nR, non‐responder; PD‐1, programmed cell death protein 1; R, responder

Studies identified CD4^+^ T cells as crucial for ICI response.^17^ However, we found that responders and non‐responders had comparable percentages of CD4^+^ T cells (Figure 2A, *p* = 0.6274). Of note, responders tended to have less CD8^+^ T cells in the peripheral blood than non‐responders (*p* = 0.3274). Since ICI therapies target the checkpoint molecules CTLA‐4 and PD‐1, we next investigated their expression on T cells. Responders tended to have lower median percentages of CTLA‐4^+^ and PD‐1^+^ CD4^+^ and CD8^+^ T cells than non‐responders (Figure 2B,C, *p*(CTLA‐4^+^ CD8^+^ T cells) = 0.0615). Of note, responders had significantly less CD4^+^CTLA‐4^+^ T cells than non‐responders had (*p* = 0.0319 *). It is known that T cells upregulate CTLA‐4 and PD‐1 on their cell surface as a negative feedback loop prior to T‐cell proliferation or T‐cell exhaustion. ICI resistance has been associated with T‐cell exhaustion, which manifests as (i) multiple co‐inhibitory checkpoint molecule presentation (PD‐1, CTLA‐4), (ii) low proliferation (Ki‐67), and (iii) impaired IFN‐γ signaling.^18^ This indicates that T cells in responders were less exhausted prior to ICI treatment than in non‐responders. Indeed, responders had significantly higher percentages of proliferating Ki‐67^+^CD4^+^ T cells than non‐responders (Figure 2D, *p* = 0.0420 *). Similar tendencies could be observed for CD8^+^ T cells (*p* = 0.2065). Data indicate that CD8^+^ T‐cell proliferation was ten times higher than CD4^+^ T‐cell proliferation. Cancer antigen–specific cytotoxic CD8^+^ T cells detect and lyse cancer cells by releasing cytolytic proteins, such as granzyme B.^19^ We could not measure significant differences in effector cytokine levels (IFN‐γ, granzyme B) in T cells between responders and non‐responders (data not shown). Except for the data on IFN‐γ, our findings suggest that non‐responders had more exhausted T cells than responders had before the start of ICI therapy.

To examine in more detail how T‐cell exhaustion parameters before the start of therapy affected therapy outcome and OS, the non‐responder group was split into two groups based on their designated cutoff. Data indicate that non‐responders benefited from low CD4^+^, CTLA‐4^+^CD4^+^, and PD‐1^+^CD8^+^ T‐cell percentages (Figure 2E, Figure S1B). Non‐responders with CTLA‐4^+^CD4^+^ and PD‐1^+^CD8^+^ T cells above the cutoff, indicative of exhausted T cells, had two thirds and half the 1‐year OS than non‐responders below the cutoffs (Figure 2E; Figure S1B). In CD8^+^ T cells, high PD‐1 percentages decreased the OS of non‐responders greater than high CTLA‐4 percentages (Figure S1B).

In short, we found that responders to ICI therapy showed an abundance of proliferating CD4^+^ T cells, while T cells in non‐responders proliferated less and expressed higher percentages of the checkpoint molecules CTLA‐4 and PD‐1. Non‐responders showed more signs of T‐cell exhaustion (low proliferation, high checkpoint molecule percentages) than responders. These findings highlight that proliferating unexhausted CD4^+^ T cells prior to therapy may be crucial for ICI therapy response.

### 3.4 Low CTLA‐4^+^ regulatory T‐cell (Treg) percentages indicate ICI therapy response

Activated GARP^+^ (glycoprotein A repetitions predominant) Treg suppress T‐cell proliferation via GARP^20^ and TGF‐β^21^ as well as impede T‐cell activation via CTLA‐4‐mediated trans‐endocytosis of the co‐stimulatory receptor CD80 on dendritic cells.^22^ Treg express CTLA‐4 constitutively. Therefore, anti‐CTLA‐4 ICI, like Ipilimumab, may bind to and deplete CTLA‐4^+^ Treg via ADCC/ADCP.^7^ Since Treg may present a competitive target to anti‐CTLA‐4 therapy, we determined the percentage of CD4^+^CD25^+^CD127^low^Foxp3^+^ Treg in the peripheral blood of patients via flow cytometry.

**FIGURE 3:**
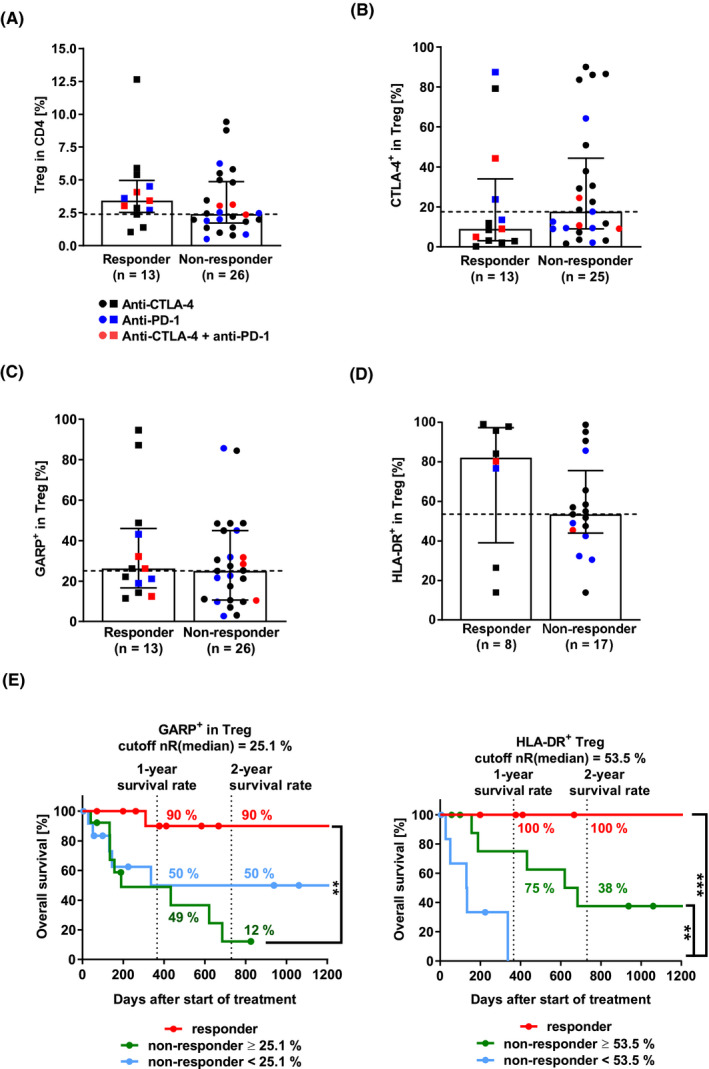
Immunomonitoring of regulatory T cells (Treg). (A) Treg. The bar diagram depicts the percentage of CD4^+^ CD25^+^CD127^low^Foxp3^+^ Treg in CD4^+^ T cells (two‐tailed Mann‐Whitney test, *p* = 0.1681). (B) CTLA‐4^+^ Treg (*p* = 0.2101). (C) GARP^+^ Treg (*p* = 0.4525). (D) HLA‐DR^+^ Treg (*p* = 0.2154). (E) Survival analysis of patients depending on GARP^+^ and HLA‐DR^+^ Treg (Log‐rank (Mantel‐Cox) test, GARP^+^ Treg, *p*(nR >25.1% vs. R) = 0.0011 **; HLA‐DR^+^ Treg, *p*(nR >53.5% vs. nR <53.5%) = 0.0069 **, *p*(nR <53.5% vs. R) = 0.0006 ***). Median with interquartile range. CTLA‐4, cytotoxic T‐lymphocyte–associated protein 4; Foxp3, forkhead protein 3; GARP, glycoprotein A repetitions predominant; HLA‐DR, human leukocyte antigen‐DR isotype; nR, non‐responder; PD‐1, programmed cell death protein 1; R, responder; Treg, regulatory T cells

Data show that the median Treg percentage in responders was one third higher than in non‐responders (Figure 3A, *p* = 0.1681). Although this difference was not significant, we also investigated Treg function and activation status. Responders had half the percentage of CTLA‐4^+^ Treg than non‐responders (Figure 3B, *p* = 0.2101) but comparable percentages of peripheral activated GARP^+^ Treg (Figure 3C, *p* = 0.4525). Treg with strong HLA‐DR expression is discussed to have a strong immunosuppressive capacity.^23^ We found that the median percentage of HLA‐DR^+^ Treg in the peripheral blood of responders was 35% higher than in non‐responders (Figure 3D, *p* = 0.2154). Interestingly, these data indicate that responders to ICI therapy may have peripheral Treg with stronger immunosuppressive capacity than non‐responders.

To quantify the T cell‐to‐Treg composition in the peripheral blood, we calculated the corresponding T cell‐to‐Treg ratios. This ratio is a widely used indicator of immune response as it indicates if Treg may overpower T cells or vice versa.^24^ Non‐responders tended to have higher CD4^+^ T cell‐to‐Treg ratios than responders (Figure S1C, *p*(CD4^+^ T cell/Treg) = 0.1681). This was due to comparable CD4^+^ T‐cell percentages in responders and non‐responders, while Treg percentages in non‐responders were lower than in responders. Additionally, we found that responders and non‐responders had comparable CD8^+^ T cell‐to‐Treg ratios (*p* = 0.8027).

In terms of OS, high percentages of peripheral GARP^+^ Treg decreased the OS of non‐responders significantly compared to responders (Figure 3E, *p* = 0.0011 **). Of note, non‐responders with peripheral HLA‐DR^+^ Treg above the cutoff lived significantly longer than non‐responders below the cutoff (*p* = 0.0069 **). T cell‐to‐Treg ratios above the cutoff reduced the 1‐year OS of non‐responders by about one third (Figure S1D).

To sum up, we found that responders tended to have higher Treg, higher HLA‐DR^+^ Treg, and lower CLTA‐4^+^ Treg levels in the peripheral blood than non‐responders, while both had similar GARP^+^ Treg percentages. Data indicate that non‐responder may benefit from low T cell‐to‐Treg ratios in the peripheral blood in terms of OS.

### 3.5 Low PMN‐MDSC and arginase 1^+^ PMN‐MDSC levels lead to ICI therapy response

Similar to Treg, MDSC suppress T‐cell activation and proliferation; one such way is by releasing the effector molecule arginase 1.^25^ Since we found that non‐responders had twice the number of monocytic cells (which also include MDSC) than responders, which may be involved in resistance to ICI therapy, we measured (arginase 1)^+^ MDSC in patient PBMC via flow cytometry (Figure S2A).

**FIGURE 4:**
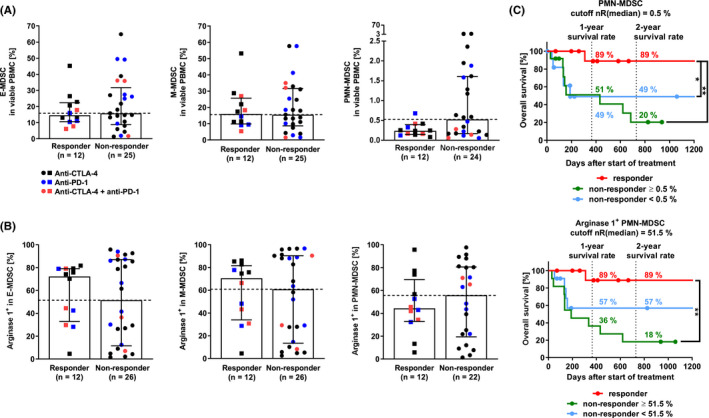
Immunomonitoring of myeloid‐derived suppressor cells (MDSC). Analysis distinguished CD15^−^CD14^+^CD33^high^HLA‐DR^low^ mononuclear/monocytic MDSC (M‐MDSC), CD15^+^CD33^+^ polymorphonuclear MDSC (PMN‐MDSC), and HLA‐DR^low^CD11b^+^CD33^+^ early MDSC (E‐MDSC). The MDSC gating strategy is depicted in Figure S2A. (A) MDSC (two‐tailed Mann‐Whitney test, *p*(E‐MDSC) = 0.6658, *p*(M‐MDSC) = 0.9872, *p*(PMN‐MDSC) = 0.1042). (B) Arginase 1^+^ MDSC (*p*(arginase 1^+^ E‐MDSC) = 0.9630, *p*(arginase 1^+^ M‐MDSC) = 0.8405, *p*(arginase 1^+^ PMN‐MDSC) = 0.7900). (C) Survival analysis of patients depending on (arginase 1^+^) PMN‐MDSC (Log‐rank (Mantel‐Cox) test, PMN‐MDSC, *p*(nR >0.5% vs. R) = 0.0064 **, *p*(nR <0.5% vs. R) = 0.0422 *; arginase 1^+^ PMN‐MDSC, *p*(nR >51.5% vs. R) = 0.0018 **). Median with interquartile range. CTLA‐4, cytotoxic T‐lymphocyte–associated protein 4; E‐MDSC, early MDSC; HLA‐DR, human leukocyte antigen‐DR isotype; M‐MDSC, monocytic/mononuclear myeloid‐derived suppressor cells; nR, non‐responder; PD‐1, programmed cell death protein 1; PMN‐MDSC, polymorphonuclear MDSC; R, responder

Whereas responders had half the PMN‐MDSC levels of non‐responders (Figure 4A, *p* = 0.1042), responders and non‐responders had comparable levels of E‐MDSC and M‐MDSC (*p*(E‐MDSC) = 0.6658, *p*(M‐MDSC) = 0.9872). Regarding tumor stage, our data confirmed that patients with stage IV melanoma tended to have higher E‐MDSC and M‐MDSC levels than healthy donors^26^, ^27^ (Figure S2B, *p*(E‐MDSC) = 0.4124, *p*(M‐MDSC) = 0.2679). However, healthy donors had higher PMN‐MDSC levels than patients with stage IV melanoma (*p* = 0.5949). In this study, PMN‐MDSC levels were about 30 times lower than M‐MDSC and E‐MDSC levels (Figure 4A). This could be attributed to the measurement of thawed samples and the corresponding decrease in the PMN‐MDSC marker CD15 in the process.^28^ To evaluate MDSC percentages based on immunosuppressive capacity, we measured the L‐arginine‐converting enzyme arginase 1 in MDSC. Arginase 1 creates a shortage of L‐arginine, which is essential for CD3ζ chain production and therefore may decrease T‐cell proliferation. Responders tended to have lower arginase 1^+^ PMN‐MDSC levels than non‐responders (*p* = 0.7900), whereas arginase 1^+^ E‐MDSC and M‐MDSC levels in responders were higher than in non‐responders (Figure 4B, *p*(arginase 1^+^ E‐MDSC = 0.9630), *p*(arginase 1^+^ M‐MDSC) = 0.8405). These data suggest that low PMN‐MDSC and arginase 1^+^ PMN‐MDSC levels lead to ICI therapy response.

In terms of OS, non‐responders with PMN‐MDSC and arginase 1^+^ E‐MDSC, M‐MDSC, and PMN‐MDSC below the cutoff had more than twice the 2‐year OS than non‐responders above the cutoff (Figure 4C; Figure S2C).

In conclusion, data indicate that low PMN‐MDSC and arginase 1^+^ PMN‐MDSC levels may be crucial for ICI therapy response. In addition, results indicate that low PMN‐MDSC and arginase 1^+^ E‐MDSC, M‐MDSC, and PMN‐MDSC levels support long‐term OS of non‐responders.

## 4 DISCUSSION

In the present study, we investigated how the immune signature of the peripheral blood prior to immune checkpoint inhibitor (ICI) therapy differs in responders and non‐responders. Notably, non‐responders with an immune signature comparable to responders displayed a prolonged overall survival (OS). In this study, the 2‐year OS of responders to ICI therapy was three times higher than that of non‐responders, underlining the importance of elucidating differences in immune cell composition in the peripheral blood which can affect ICI therapy response (Table 2).

**TABLE 2:** Summary of results—CBC and immune cell composition in the peripheral blood of patients with malignant melanoma before the beginning of ICI therapy

Parameter	Responder (R)	Non‐responder (nR)
Complete blood count, median		
LDH, units/L	220	333
S100, µg/L	0.1	0.1
Platelet count, counts/nl	241	253
Platelet‐to‐lymphocyte ratio	135	289
Lymphocytes and Monocytes, median %		
Lymphocytes	69	50
Monocytes	15	29
Lymphocyte‐to‐monocyte ratio	5	1.6
Regulatory T cells (Treg), median %		
Treg	3	2
CTLA‐4	9	18
GARP	26	25
HLA‐DR	82	54
T cells, median %		
CD4^+^ T cells	63	63
Ki‐67	1.2	0.4
CTLA‐4	6	14
PD‐1	16	22
T cell‐to‐Treg ratio	29	43
CD8^+^ T cells	15	19
Ki‐67	28	15
CTLA‐4	1	2
PD‐1	16	21
T cell‐to‐Treg ratio	10	10
Myeloid‐derived suppressor cells (MDSC), median %		
E‐MDSC	16	16
Arginase 1	71	52
M‐MDSC	15	16
Arginase 1	72	61
PMN‐MDSC	0.2	0.5
Arginase 1	44	56

Note

Table 2 summarizes the medians of measured parameters in responders and non‐responders to ICI therapy.

Abbreviations: CTLA‐4, cytotoxic T‐lymphocyte–associated protein; E‐MDSC, early MDSC; GARP, glycoprotein A repetitions predominant; HLA‐DR, human leukocyte antigen‐DR isotype; ICI, immune checkpoint inhibitor; LDH, lactate dehydrogenase; MDSC, myeloid‐derived suppressor cells; M‐MDSC, monocytic/mononuclear MDSC; PD‐1, programmed death receptor‐1; PMN‐MDSC, polymorphonuclear MDSC; S100, S100 protein.

We found that responders had high levels of lymphocytes, which mainly consisted of proliferating CD4^+^ T cells, whereas T cells in non‐responders displayed a tendency toward an exhausted T‐cell phenotype, such as Ki‐67^low/−^CTLA‐4^+^/PD‐1^+^ T cells. Responder complete blood count reported a lower platelet count, PLR, and, in accordance with literature, a lower level of the melanoma biomarkers, LDH and S100, compared to non‐responders. Responders had lower levels of CTLA‐4^+^ Treg, PMN‐MDSC, and arginase 1^+^ PMN‐MDSC than non‐responders. Notably, immune cell composition also positively affected the long‐term OS of a subgroup of “clinical” non‐responders. Herein, this subgroup displayed long‐term OS with low LDH, platelet count, PLR, CTLA‐4^+^CD4^+^ T cells, HLA‐DR^+^ Treg, and (arginase 1^+^) PMN‐MDSC levels comparable to responders.

A strong T‐cell response before beginning ICI therapy is associated with better outcome.^29^, ^30^, ^31^ In line with this, we found high percentages of proliferating (Ki‐67^+^) T cells and a CTLA‐4^−^/PD‐1^−^ T cell phenotype in responders compared to non‐responders. This was significant for Ki‐67^+^CD4^+^ T cells. Since we analyzed T‐cell proliferation (Ki‐67) in thawed CD3^+^CD4^+^ peripheral mononuclear cells (PBMC) without prior stimulation, the percentage of Ki‐67^+^CD4^+^ T cells was lower in comparison with data from stimulated PBMC or memory phenotype CD4^+^ T cells.^32^, ^33^ High percentages of proliferating (Ki‐67^+^) CD4^+^ T cells in responders of ICI therapy are consistent with Simpson et al.'s observation that CD4^+^ T cells were crucial mediators of the anti‐CTLA‐4 mechanism of action in a B16‐BL6 melanoma mouse model.^6^ Furthermore, Arakawa et al. reported that the TCR repertoire of CD4^+^ T cells in patients increased after the start of anti‐CTLA‐4 ICI therapy but not in CD8^+^ T cells.^29^ Arakawa et al. associated a broad TCR repertoire (richness) with long‐term OS. However, we found that non‐responders with high CD4^+^ T‐cell percentages before the beginning of therapy tended to have a lower probability of long‐term OS. This raises the question of whether the TCR repertoire of CD4^+^ T cells in these non‐responders was low and, therefore, resulted in a lower OS. This should be analyzed in future studies.

ICI release the brakes on T‐cell proliferation but do not directly activate T cells. We suggest that patients may become non‐responders to ICI therapy due to having either (i) T cells that are too exhausted to respond, (ii) not activated T cells that respond slowly/late (no prior proliferation to release the brakes on), or (iii) activated (not tumor‐specific) T cells that may lead to strong immune‐related adverse events (irAE) and thus the discontinuation of therapy. Therefore, we theorize that patients with proliferating tumor‐specific T cells (at baseline) may become responders to ICI therapy. It is known that response to ICI therapy in patients with melanoma correlates with high tumor mutational burden, neoantigen load,^34^ and MHC‐II expression.^35^ Regarding neoantigen load, we know that combined BRAF/MEK inhibitor treatment can induce apoptosis in BRAF‐mutated melanoma and, hence, trigger the release of possible tumor‐restricted antigens.^36^ These neoantigens could increase tumor immunogenicity and facilitate a tumor‐specific T‐cell response.^37^ In a BRAF‐mutated melanoma, this T‐cell response could be amplified, by broadening the TCR repertoire at baseline (BRAF/MEK inhibitors) and by subsequently increasing the number of proliferating CD4^+^ T cells (checkpoint inhibitors), thus improving the likelihood of a positive ICI therapy response.

Previous studies support the idea that CD8^+^ T cells play a key role in the anti‐tumor immune response and that their presence in the tumor microenvironment (TME) is a beneficial prognostic indicator.^38^ Although our results displayed reduced frequencies of CD8^+^ T cells in responders compared to non‐responders, it is important to note that these results come from sampling patient peripheral blood before treatment rather than from the tumor itself. Spassova et al. described in Merkel cell carcinoma that resistance to ICI can also be attributed to insufficient influx of CD8^+^ T cells into the tumor and a loss of MHC‐I expression.^39^ We speculate that high CD8^+^ T cell percentages in the peripheral blood of non‐responders were increased due to reduced or impaired migration into the TME. Reduced CD8^+^ T‐cell levels in the peripheral blood of responders could be indicative of a greater influx of CD8^+^ T cells into the TME. Nevertheless, future studies must be conducted to compare relative levels of CD8^+^ T cells in patient peripheral blood and tumors simultaneously to further support this idea.

The TME induces a regulatory (Treg) and immature tolerogenic (MDSC) phenotype in immune cells, recruits, and expands these cell populations not only in the TME but also in the peripheral blood.^40^ Consistent with this, we found that E‐MDSC and M‐MDSC frequencies in melanoma patients were increased compared to healthy donors.^41^ In this study, high percentages of immunosuppressive Treg and MDSC were contradictory regarding ICI response and long‐term OS. We found that low (arginase 1^+^) PMN‐MDSC and CTLA‐4^+^ Treg indicated ICI therapy response. Since CTLA‐4^+^ Treg may impede T‐cell activation via trans‐endocytosis of the co‐stimulatory receptor CD80 on dendritic cells,^22^ low percentages of CTLA‐4^+^ Treg in responders suggest that responders harbor greater numbers of dendritic cells to activate T cells than non‐responders. In addition, low (arginase 1^+^) PMN‐MDSC levels in responders may also favor stronger T‐cell activation. In agreement with our data, Meyer et al. found this to be the case for low levels of circulating M‐MDSC in melanoma patients undergoing Ipilimumab treatment.^41^ Future studies should investigate the wide variety of MDSC subpopulations in the peripheral blood in more detail to clarify these findings. Rudolph et al. highlighted the disadvantageous role of MDSC because they found that CD11b^+^CD33^+^CD14^+^HLA‐DR^low^ M‐MDSC correlated positively with melanoma stages.^27^ We confirmed this observation for M‐MDSC and expanded it to include E‐MDSC. This is important because MDSC are discussed as new therapeutic targets for cancer.^42^ MDSC targeting therapeutics could assist ICI by enhancing T‐cell proliferation. Since MDSC are also discussed to cause resistance to cancer immunotherapies, we recommend monitoring MDSC during ICI therapy to investigate MDSC‐derived resistance mechanisms.^8^ This is consistent with high percentages of arginase 1^+^ M‐MDSC, E‐MDSC as well as (HLA‐DR^+^) Treg indicating ICI therapy response. Since ICI release the brakes on T‐cell proliferation, irrespective of tumor or non–tumor‐specific T cells, and thereby lead to strong irAE, peripheral MDSC and Treg may protect patients from strong irAE in the periphery and avoid discontinuation of therapy. On the other hand, high Treg, E‐MDSC, and M‐MDSC percentages may be a feedback effect of strong T‐cell proliferation or be due to low influx of Treg into the TME in responders. As already mentioned above for CD8^+^ T effector cells, future studies must be conducted to compare relative levels of Treg in peripheral blood and the tumor simultaneously.

In this study, the immune signature of the peripheral blood affected long‐term OS of a group of “clinical” non‐responders. To the best of our knowledge, this has not been shown before in the literature and is significant when evaluating the clinical response and prognosis in melanoma patients. In this study, non‐responders with low PLT, PD‐1^+^CD8 T cells, and arginase 1^+^ PMN‐MDSC had twice the 1‐year OS compared to non‐responders with high levels. The latter is consistent with the observation that circulating MDSC impede anti‐tumor immune responses in non‐responders.^8^ However, we also found that non‐responders with high percentages of HLA‐DR^+^ Treg in the blood lived significantly longer than non‐responders with low percentages. HLA‐DR^+^ Treg strongly suppress T‐cell proliferation and, as such, may dampen uncontrolled T‐cell responses in the periphery and counter strong irAE.^43^ Like responders, this may be also due to low influx of Treg into the TME.

The main goals of ICI therapy are to recruit T cells, invade the tumor, and create an inflamed TME.^44^ Based on our findings and discussion, we suggest that ICI therapy in combination with BRAF/MEK inhibitors or MDSC‐targeted therapeutics may improve T‐cell recruitment. Since responders had lower platelet counts than non‐responders, we recommend checking platelet count via complete blood count before beginning immunotherapy. Platelet counts in a normal range are important for physiological blood circulation and thus, immune cell and ICI distribution; they may also improve TME invasion and tumor inflammation. In fact, dense platelet distribution is discussed as a factor of poor prognosis in melanoma.^14^ Anti‐coagulant therapies, like low‐dose acetylsalicylic acid, might be an easy and affordable way to raise the probability of clinical efficacy of ICI therapy. To predict ICI therapy response, it would be feasible to calculate a patient score indicating the probability of ICI therapy response depending on cellular and molecular parameters.

Being aware that this study represents a discovery cohort with a relatively small number of patients, our data integrate findings, some of which are original while others are confirmatory. Nevertheless, our study provides a comprehensive overview of the immune signature influencing response or non‐response in ICI‐treated patients. Some of the data trends (responders vs. non‐responders) may be valuable but remain insignificant due to the limited sample size. Parameters like the lymphocyte‐to‐monocyte ratio may be of practical importance and should be further validated in an expanded study. Of note, this study population does not represent today's treatment guidelines for ICI. During the recruitment phase of the study (April 2014–July 2017), the therapeutic guidelines for the treatment of unresectable, late‐stage, malignant melanoma changed. Instead of anti‐CTLA‐4 monotherapy (recommended from February 2013 to July 2016), the new guidelines recommend anti‐PD‐1 or anti‐PD‐1/CTLA‐4 combination therapy.^45^, ^46^ Since a major part of the study cohort was recruited before July 2016, Ipilimumab is the predominant treatment in this study. We propose to confirm our results in a larger validation cohort with equal size ICI treatment groups. We recommend analyzing the anti‐CTLA‐4, anti‐PD‐1, and anti‐CTLA‐4/PD‐1 therapy groups separately because expression levels of CTLA‐4 and PD‐1 differed between immune cells, varied in effect on OS, and, as is known, initiate different mechanisms of action (i.e., possible Treg depletion under anti‐CTLA‐4).

In conclusion, our study demonstrated that immune cell composition in the peripheral blood of melanoma patients at baseline affects ICI therapy response and long‐term OS in a group of non‐responders thus benefiting from ICI therapy. Responders displayed (i) low platelet‐to‐lymphocyte ratios, (ii) increased lymphocyte‐to‐monocyte ratios, (iii) increased proliferating CTLA‐4^−^, PD‐1^−^ CD4^+^ T cells, (iv) low CTLA‐4^+^ Treg, (v) low PMN‐MDSC, and (vi) arginase 1^+^ PMN‐MDSC compared to non‐responders. Importantly, non‐responders to ICI therapy with similar characteristics showed long‐term OS. Using this immune signature may present a practical and valuable approach to identify patients prior to treatment that will best respond to and thus benefit from ICI immunotherapy. Notably, we discovered a novel group of non‐responders that had a similar immune signature to responders and had a longer overall survival.

## CONFLICT OF INTEREST

The authors state no conflict of interest.

## Supporting information

Fig S1

Fig S2

Table S1

## Data Availability

The data that support the findings of this study are available from the corresponding author upon reasonable request.
