# Supplementary figures and images for: Immune signature as predictive marker for response to checkpoint inhibitor immunotherapy and overall survival in melanoma
Source: Cancer Med. 2021 Jan 15;10(5):1562–75. doi: 10.1002/cam4.3710 (PMC7940230; doi:10.1002/cam4.3710)

Supplementary Figure S1.

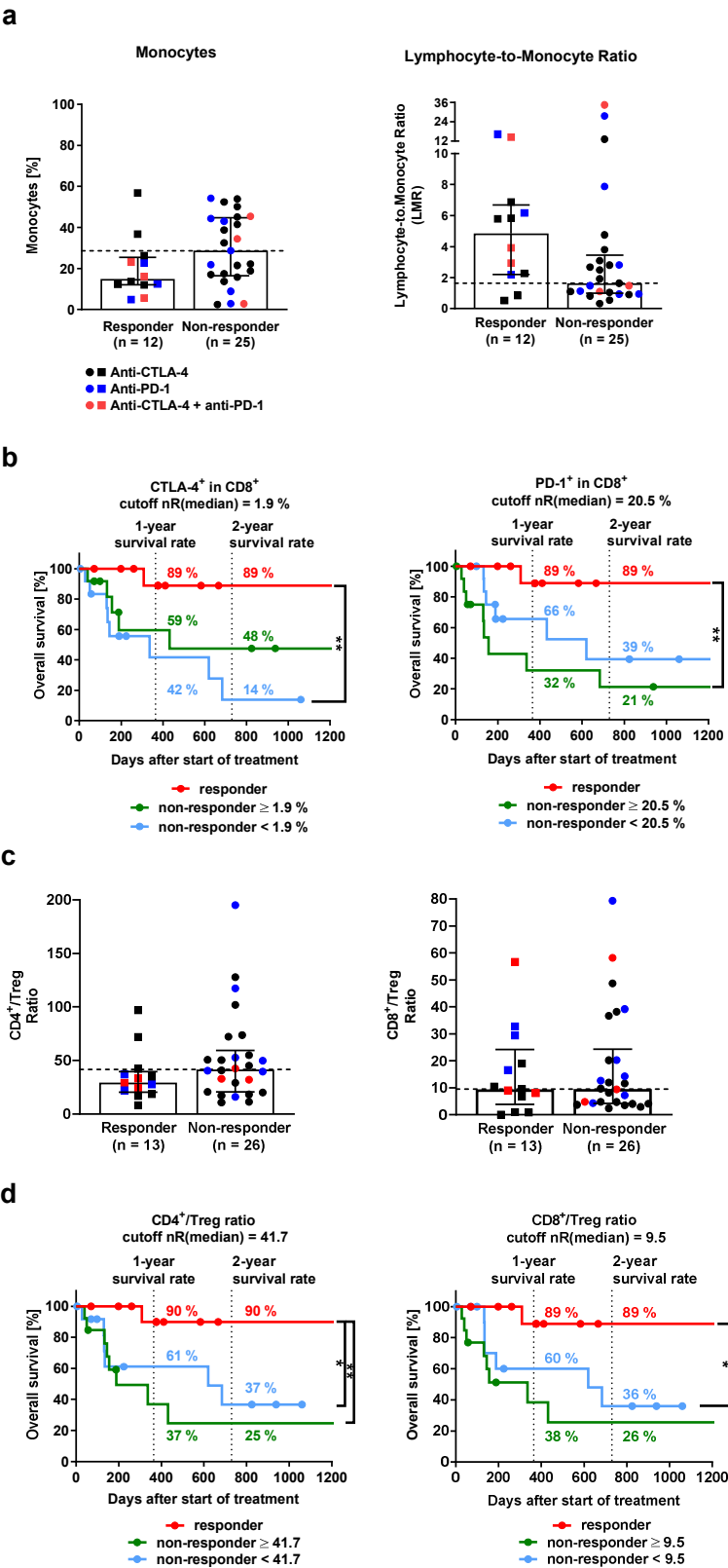

Supplement: Supplementary file 1 — Fig S1 [file CAM4-10-1562-s001.pdf]

Supplementary Figure S2.

**a**

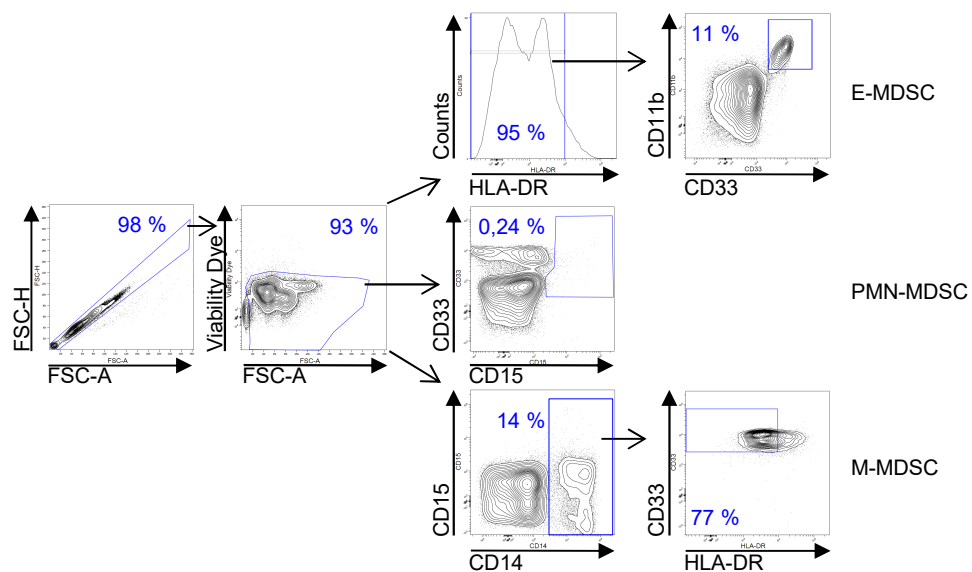

**b**

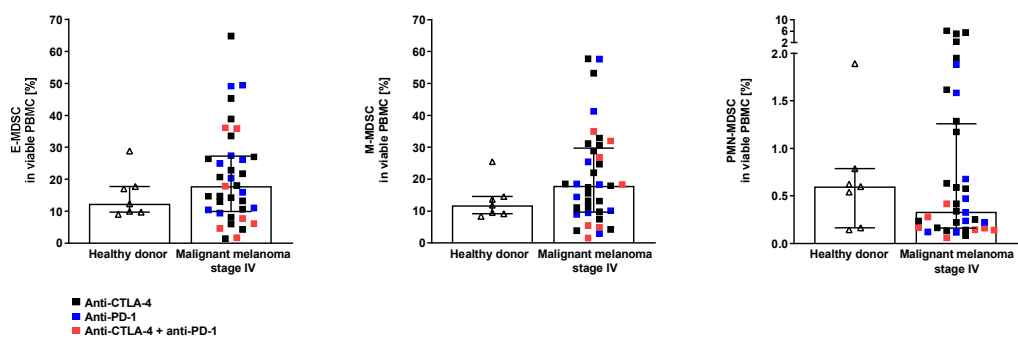

**c**

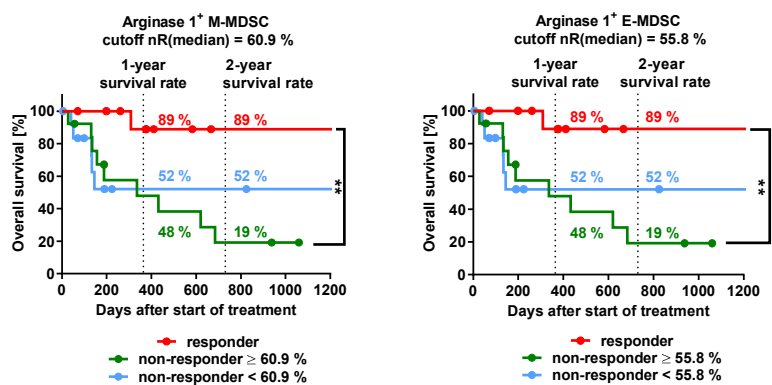

Supplement: Supplementary file 2 — Fig S2 [file CAM4-10-1562-s003.pdf]
