# Supplementary material for: Immune signature as predictive marker for response to checkpoint inhibitor immunotherapy and overall survival in melanoma
Source: Cancer Med. 2021 Jan 15;10(5):1562–75. doi: 10.1002/cam4.3710 (PMC7940230; doi:10.1002/cam4.3710)
Supplement: Supplementary file 3 — Table S1 [file CAM4-10-1562-s002.pptx]

## Slide 1
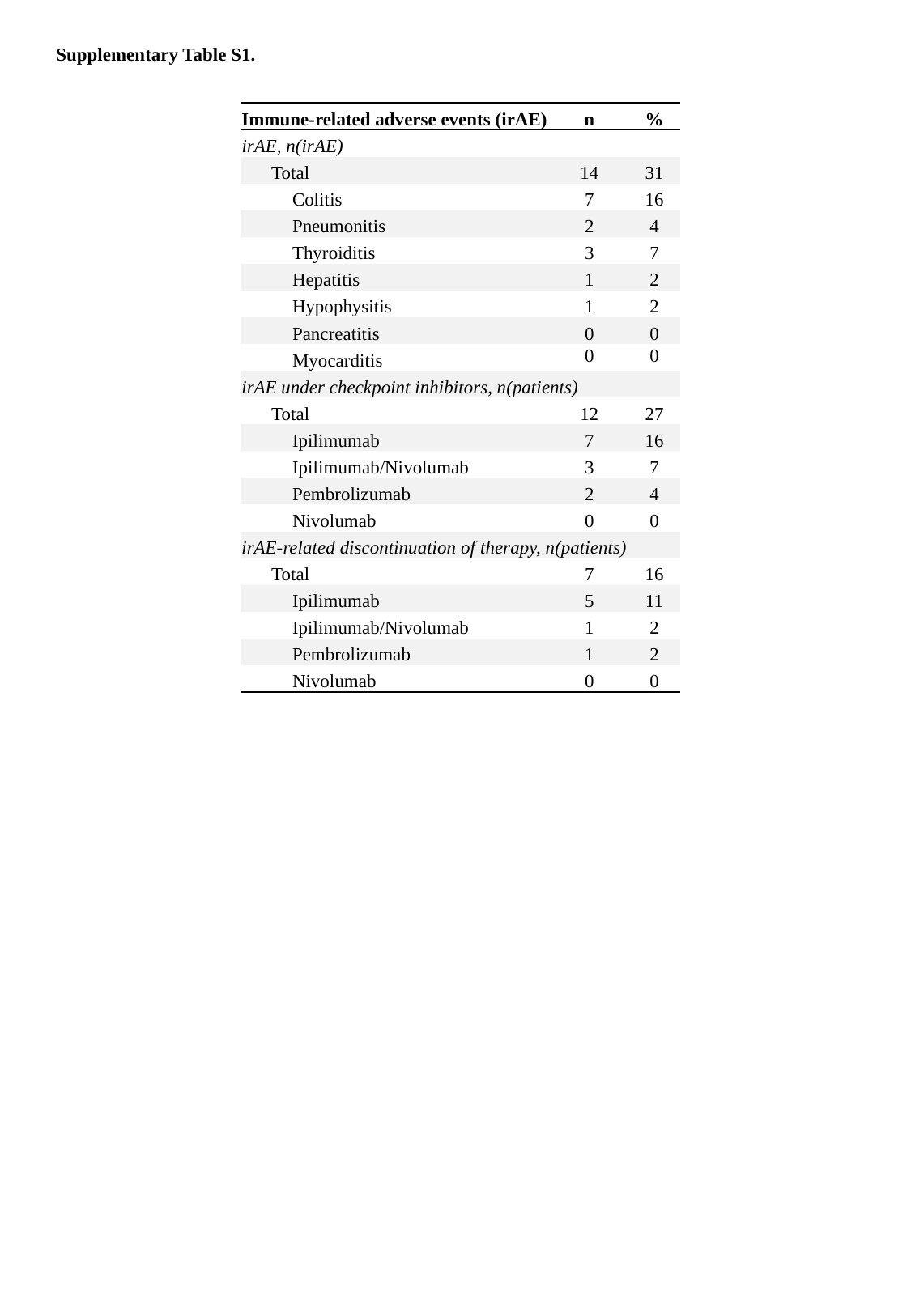

Supplementary Table S1.
| Immune-related adverse events (irAE) | n | % |
| --- | --- | --- |
| irAE, n(irAE) | | |
| Total | 14 | 31 |
| Colitis | 7 | 16 |
| Pneumonitis | 2 | 4 |
| Thyroiditis | 3 | 7 |
| Hepatitis | 1 | 2 |
| Hypophysitis | 1 | 2 |
| Pancreatitis | 0 | 0 |
| Myocarditis | 0 | 0 |
| irAE under checkpoint inhibitors, n(patients) | | |
| Total | 12 | 27 |
| Ipilimumab | 7 | 16 |
| Ipilimumab/Nivolumab | 3 | 7 |
| Pembrolizumab | 2 | 4 |
| Nivolumab | 0 | 0 |
| irAE-related discontinuation of therapy, n(patients) | | |
| Total | 7 | 16 |
| Ipilimumab | 5 | 11 |
| Ipilimumab/Nivolumab | 1 | 2 |
| Pembrolizumab | 1 | 2 |
| Nivolumab | 0 | 0 |
